# Differences in psychosocial distress among rural and metropolitan health care workers during the COVID‐19 pandemic

**DOI:** 10.1111/ajr.12873

**Published:** 2022-05-05

**Authors:** Rachel Tham, Amy Pascoe, Karen Willis, Margaret Kay, Natasha Smallwood

**Affiliations:** ^1^ Allergy and Lung Health Unit, Centre for Epidemiology and Biostatistics, Melbourne School of Population and Global Health The University of Melbourne Melbourne Victoria Australia; ^2^ La Trobe Rural Health School La Trobe University Bendigo Victoria Australia; ^3^ Mary MacKillop Institute for Health Research Australian Catholic University Melbourne Victoria Australia; ^4^ Department of Allergy, Immunology and Respiratory Medicine, Central Clinical School The Alfred Hospital, Monash University Melbourne Victoria Australia; ^5^ College of Health and Biomedicine Victoria University Melbourne Victoria Australia; ^6^ Division of Critical Care and Investigative Services Royal Melbourne Hospital Parkville Victoria Australia; ^7^ Primary Care Clinical Unit Royal Brisbane and Women's Hospital Herston Queensland Australia; ^8^ Department of Respiratory Medicine The Alfred Hospital Prahran Victoria Australia

**Keywords:** COVID‐19, health care workers, mental health, psychosocial stress, well‐being

## Abstract

**Objective:**

The Australian COVID‐19 Frontline Healthcare Workers study examined the prevalence and severity of mental health symptoms during the second wave of the COVID‐19 pandemic. This substudy examined the differences in psychological well‐being between rural and metropolitan health care workers (HCWs).

**Design:**

A nationwide survey conducted between August and October 2020.

**Setting and Participants:**

Australian HCWs were recruited through multiple strategies.

**Main outcome measures:**

Demographics, mental health outcomes (anxiety, depression, post‐traumatic stress disorder [PTSD] and burnout).

**Results:**

Complete responses were included from 7846 participants, with 1473 (18.8%) in regional or remote (‘rural’) areas and 81.2% in metropolitan areas. Rural participants were older, more likely to work in allied health, nursing or in health administration, and had worked longer in their profession than metropolitan participants. Levels of resilience were similar (*p* = 0.132), but there was significantly higher prevalence of pre‐COVID‐19 pandemic mental illness in the rural workforce (*p* < 0.001). There were high levels of current mental health issues: moderate–severe PTSD (rural 38.0%; metropolitan 41.0% *p* = 0.031); high depersonalisation (rural 18.1%; metropolitan 20.7% *p* = 0.047); and high emotional exhaustion (rural 46.5%; metropolitan 43.3% *p* = 0.002). Among rural participants, mental health symptoms were associated with younger age, worry about being blamed if they contracted COVID‐19, fear of transmitting COVID‐19 to their family, experiencing worsening relationships and working in primary care or allied health.

**Conclusion:**

Despite having low COVID‐19 case numbers in rural Australian health services compared with metropolitan counterparts over the course of 2020, there were widespread mental health impacts on the workforce. Rural health services need specific and flexible training, education, work policies and practices that support psychological well‐being now in preparedness for ongoing or future crises.

**What is already known on this subject:**

- Health care workers experience distinctive workplace demands and stress and are at increased risk of poorer mental health
- Pandemics bring additional threats to the mental well‐being of health care workers
- Compromised health care worker mental health has implications for patient safety, quality of care and workforce retention

**What does this study add:**

- The COVID‐19 pandemic has adversely impacted the mental health of Australian health care workers in rural and metropolitan Australia
- Rural health care workers experienced a high prevalence of mental health symptoms despite treating very few COVID‐19 patients during this period
- Rural health care services need to be prepared for the mental health impacts of COVID‐19 within their workforce

## 1 Introduction

Health care workers (HCWs) experience unique workplace stress, which contribute to increased rates of occupational burnout, anxiety, depression, stress and poorer mental health.^1^, ^2^, ^3^, ^4^ Health‐related crises, such as the current SARS‐CoV2 coronavirus disease (COVID‐19) pandemic, generate significant disruption and occupational stress. Globally, the workplace challenges arising from COVID‐19 have been rapid and varied, including surge demand on health care services, increased workloads, new and more complex work practices, large amounts of new information, job insecurity, major social changes and increased risks to the health and lives of HCWs and those close to them. ^5^, ^6^ International data have demonstrated that HCWs have experienced high levels of stress, anxiety, depression, moral distress and burnout during the pandemic.^7^, ^8^, ^9^

Australia's suppression strategy for managing COVID‐19 spread has resulted in low levels of community transmission and low mortality compared to the international experience.^10^ Nevertheless, the Australian COVID‐19 Frontline Healthcare Workers Study, which included almost ten thousand respondents, identified high levels of occupational and social disruption, anxiety, depression, moral distress and burnout among frontline HCWs during the pandemic.^5^, ^11^, ^12^, ^13^, ^14^ These effects were not related to caseload of COVID‐19 patients and were experienced differentially across age groups, genders and health disciplines with more adverse impacts reported in younger groups, or among female participants or those who worked in nursing.

Importantly, HCWs might also be differentially impacted by the pandemic depending on whether they practice in rural, regional, remote or metropolitan settings. Practising in these different locations is associated with different stress.^15^ In the Australian health care system rural, regional and remote (simplified as ‘rural’ for this paper) areas are classified in geographic terms according to their population size, road distance to service centres (accessibility), and access and equity indicators associated with health workforce recruitment and retention.^16^ Metropolitan areas include most capital cities (except Darwin) and cities with populations over 200 000 people.^16^ As populations in rural settings are smaller, there are fewer hospitals that provide onsite radiology and pathology, specialised emergency care and limited intensive care facilities (if any) for severely unwell people.^17^In rural Australia, there is greater emphasis on the provision of integrated primary health care, outreach services and general practitioner (GP)‐based hospitals in response to local geographic and cultural needs.^18^ Currently, little is understood about the mental health impacts of the COVID‐19 pandemic among rural HCWs compared with metropolitan HCWs.

During 2020, Australia experienced a significant internal migration with approximately 43 000 people moving from metropolitan to rural and regional areas in Australia, a trend that continued through 2021.^19^ This has serious implications for the existing rural health care system and workforce, which has not changed significantly in response to this internal migration. It is predicted that the COVID‐19 pandemic will continue for several years resulting in surge demands on health care systems across metropolitan and rural regions of Australia.^10^ In planning for the long‐term needs of the current and future health care workforce, it is crucial to identify the prevalence of, and risk factors associated with, mental health problems experienced by Australian HCWs working in rural regions and understand how they differ from metropolitan regions.

This paper reports a subset of findings from the Australian COVID‐19 Frontline Healthcare Workers Study. It focuses on the association between practice location (rural or metropolitan) and the severity and factors associated with psychosocial stress. We hypothesised that the prevalence and predictors of mental health symptoms would vary according to practice location.

## 2 Methods

### 2.1 Study design and sample

The full study methodology has been published.^11^ In brief, a nationwide, voluntary, anonymous, online survey was conducted between 27 August and 23 October 2020, concurrently with the Australian second wave of the pandemic which occurred primarily in Melbourne, Victoria. Self‐identified health care workers working at the ‘frontline’ of public and private secondary, primary or community health care across Australia were invited to participate. Participants did not need to have direct contact with people infected with COVID‐19 to take part. Information regarding the survey was widely disseminated across Australia by hospital leaders in all Victorian public hospitals and multiple hospitals around Australia; through 36 professional societies, associations, colleges and universities; government health departments and mainstream (117 newspapers and eight television and radio interviews); and 30 social media sites. No incentives were provided to participants.

### 2.2 Data collection

Data were collected at a single time‐point. Participants provided online consent to participate in the study and then completed the online survey via a direct link or through a purpose‐built website (https://covid‐19‐frontline.com.au/). Data were collected and managed using REDCap electronic data capture tools.^20^ Information collected included demographics, professional background and workplace, the impact of the pandemic on employment and finances, organisational leadership, occupational change, health and recreational habits, mental health help‐seeking behaviours, self‐reported mental health issues (subjectively determined) and five validated, objective mental health symptom measurement tools. Five validated psychological measurement tools were used to assess anxiety (generalised anxiety disorder [GAD‐7]), depression (Patient Health Questionnaire [PHQ‐9]), post‐traumatic stress disorder (PTSD; abbreviated Impact of Event Scale [IES‐6]) and burnout (abbreviated Maslach Burnout Inventory [MBI]: subdomains of depersonalisation, emotional exhaustion and personal accomplishment). Burnout on the MBI is indicated by higher scores on the depersonalisation and emotional exhaustion subdomains, and lower scores on the personal accomplishment scale.

For each mental health scale, validated cut‐off scores were as follows: GAD7: 0–4 = none/minimal, 5–9 = mild, 10–14 = moderate, 15–20 = severe anxiety; PHQ9: 0–4 = none/minimal, 5–9 = mild, 10–14 = moderate, 15–19 = moderately severe, 20–27 = severe; IES: 0–9 = none/minimal, >9 = moderate to severe; MBI depersonalisation: 0–3 = low, 4–6 = moderate, 7–18 = high; emotional exhaustion: 0–6 = low, 7–10 = moderate, 11–18 = high; and personal accomplishment: 0–12 = high, 13–14 = moderate, 15–18 = low. From a clinical outcome perspective, we were most interested in moderate to severe impacts compared with minimal or low impacts, so outcomes were dichotomised while maintaining the validated cut‐off scores: GAD7: 0–9 = none to mild, 10–21 = moderate to severe;^21^ PHQ9: 0–9 = none to mild, 10–27 = moderate to severe;^22^ IES: 0–9 = minimal to none, >9 = moderate to severe;^23^ MBI depersonalisation: 0–3 = low, 4–18 = moderate to high; emotional exhaustion: 0–6 = low, 7–18 = moderate to high; and personal accomplishment: 0–13 = low, 13–18 = moderate to high.^24^

Resilience was assessed using the abbreviated 2‐item Connor‐Davidson Resilience Scale (CD‐RISC‐2).^25^ Further detail about the questionnaire design has been published elsewhere.^11^

Ethics approval was provided for the study by the Royal Melbourne Hospital Human Research Ethics Committee (HREC/67074/MH‐2020).

### 2.3 Statistical analysis

A power calculation was computed using R Version 4.0.4 (Vienna Australia). For an expected medium to large effect size with a power of 0.95 and significance level of 0.05, a sample of 6348 respondents was required. A minimum sample size of 7000 responses was set to account for missing or incomplete data. Demographic and socioeconomic characteristics are reported descriptively. Individual outcomes examined included objective mental health symptoms (measured on validated scales). Chi‐squared and t‐tests were used for categorical and continuous data comparisons between rural and metropolitan groups. A priori factors associated with mental health outcomes were identified, and if they were statistically significant in the univariate logistic regression models, they were then entered into multivariate logistic regression models. Associations are presented as odds ratios (OR) with 95% confidence intervals (CI). Variables examined in regression models for mental health outcomes included age, sex, state, profession, number of years working since graduating, living alone, living with children, living with people aged over 65, frontline area, working with COVID‐19 patients, anticipates working with COVID‐19 patients, received training to care for patients with COVID‐19, received personal protective equipment (PPE) training, close friends or relatives infected with COVID‐19, changes (closer and worse) in relationships with partner/parent or family/friends/work colleagues, changed household income, concerns regarding household income, pre‐existing mental health condition and resilience. Reference categories for variables are in Tables S1–S6. Data analysis was performed using Stata SE/15.1 (College Station, Texas) ^26^ and R Version 4.0.4 (Vienna Australia).^27^

## 3 Results

### 3.1 Demographics, workplace environment and changes

Over the 8‐week survey period, responses were provided by 9518 participants. The final study sample consisted of 7846 individuals from whom complete data were collected. Of these 1473 (18.8%) were in a rural area and 6373 (81.2%) were in a metropolitan area (Table 1). Participants in rural areas were significantly older (>40 years = 66.1%) than those in metropolitan areas (>40 years = 45.5%), were more likely to be working in allied health (rural = 21.8%; metropolitan = 19.6%) or were administrative health staff (rural = 11.6%; metropolitan = 6.0%), and had been working >10 years in their field (rural = 60.9%; metropolitan = 54.0%) but were less likely to be medically trained (rural = 24.0%; metropolitan = 33.0%).

**TABLE 1:** Participants' characteristics

Demographic characteristics	Rural *n* = 1473 *n* (%)	Metropolitan *n* = 6373 *n* (%)	*p*‐value
Age (years)			
20–30	262 (17.8)	1598 (25.1)	<0.001
31–40	370 (25.1)	1880 (29.5)	
41–50	319 (21.7)	1419 (22.3)	
>50	522 (35.4)	1476 (23.2)	
Gender			
Male	240 (16.3)	1218 (19.1)	0.059
Female	1227 (83.3)	5117 (80.3)	
Non‐binary	2 (0.1)	17 (0.3)	
Prefer not to say	4 (0.3)	21 (0.3)	
Lives alone	203 (13.8)	884 (13.9)	0.247
Living with ≥1child under 16 years of age at home	533 (36.2)	2211 (34.7)	0.431
Living with ≥1 elderly person at home	243 (16.5)	550 (8.6)	0.103
State/territory			
Victoria	1140 (77.4)	5545 (87.0)	<0.001
New South Wales	153 (10.4)	319 (5.0)	
Queensland	70 (4.8)	139 (2.2)	
Tasmania	44 (3.0)	37 (0.6)	
Western Australia	26 (1.8)	111 (1.7)	
South Australia	24 (1.6)	179 (2.8)	
Northern Territory	11 (0.7)	13 (0.2)	
Australian Capital Territory	5 (0.3)	30 (0.5)	
Health role			
Nursing	564 (38.3)	2518 (39.5)	<0.001
Medical	354 (24.0)	2103 (33.0)	
Allied health/paramedic/clinical scientist/dental	321 (21.8)	1249 (19.6)	
Other^a^	171 (11.6)	380 (6.0)	
Pharmacist	63 (4.3)	122 (1.9)	
Number of years working since graduating			
0–5	250 (21.5)	1342 (24.5)	<0.001
6–10	205 (17.6)	1172 (21.4)	
11–15	158 (13.6)	785 (14.3)	
>15	550 (47.3)	2175 (39.7)	

^a^ Other = Support, clerical, security or cleaning role.

Rural participants were more likely to work in primary or community care settings (26.9%) compared with metropolitan participants (14.6%) (Table 2). Significantly fewer rural participants experienced reduced working hours (9.6%) compared with their metropolitan counterparts (11.7%) during the pandemic. Metropolitan participants were more likely to have been redeployed to a new department for work (metropolitan = 17.3%; rural = 14.7%), whereas rural participants were just as likely to have had a change in their work role within their current department but were less confident in the new role (rural = 1.54; metropolitan = 1.59). Significantly more rural participants believed that their workplace provided active support for their mental health during the pandemic (rural = 20.4%; metropolitan = 15.3%).

**TABLE 2:** Workplace, financial and social change

	Rural *n* = 1473 *n* (%)	Metropolitan *n* = 6373 *n* (%)	*p*‐value
Frontline area			
Emergency department	210 (14.3)	936 (14.7)	<0.001
Intensive care unit	53 (3.6)	692 (10.9)	
Anaesthetics or surgery	78 (5.3)	746 (11.7)	
Medical specialty areas^a^	532 (36.1)	2573 (40.4)	
Other^b^	204 (13.9)	492 (7.7)	
Primary care and community^c^	396 (26.9)	933 (14.6)	
Changed working hours since the pandemic commenced^d^			
Increased paid hours	306 (20.8)	1328 (20.8)	0.957
Increased unpaid hours	324 (22.0)	1362 (21.4)	0.599
Decreased paid or unpaid hours	142 (9.6)	744 (11.7)	0.026
No change	774 (52.6)	3265 (51.2)	0.363
Change in household income due to COVID‐19			
Increased	170 (11.5)	650 (10.2)	<0.001
Decreased	380 (25.8)	2035 (31.9)	
No change	923 (62.7)	3688 (57.9)	
Concerned about household income since COVID‐19	423 (28.7)	1993 (31.3)	0.056
Redeployed to a new area of work	217 (14.7)	1101 (17.3)	0.019
Confidence in new area (mean [SD])^e^	1.58 (0.71)	1.54 (0.72)	0.331
Change in work role	424 (28.8)	1715 (26.9)	0.145
Confidence in new role (mean [SD])^e^	1.54 (0.78)	1.59 (0.73)	0.015
Communication received from the workplace during the pandemic has been useful and timely			
Strongly or somewhat agree	1068 (72.5)	4765 (748)	0.138
Neither agree nor disagree	161 (10.9)	640 (10.0)	
Strongly or somewhat disagree	244 (16.6)	968 (15.2)	
Believed their workplace actively supported their well‐being and mental health during the pandemic			
Strongly or somewhat agree	300 (20.4)	975 (15.3)	0.001
Neither agree nor disagree	262 (17.8)	957 (15.0)	
Strongly or somewhat disagree	911 (61.8)	4441 (69.7)	
Closer relationship with			
Partner	376 (25.5)	1890 (29.7)	0.002
Parent/family	385 (26.1)	1841 (28.9)	0.035
Friends	150 (10.2)	904 (14.2)	0.001
Colleagues	406 (27.6)	2127 (33.4)	0.001
Worse relationship with			
Partner	193 (13.1)	807 (12.7)	0.648
Parent/family	269 (18.3)	1152 (18.1)	0.867
Friends	394 (26.7)	1827 (28.7)	0.140
Colleagues	219 (14.9)	897 (14.1)	0.433
No effect on relationships	409 (27.8)	1443 (22.6)	0.001

^a^ includes general medicine, hospital aged care, respiratory medicine, infectious diseases and palliative care.

^b^ includes paramedicine, radiology, pathology and other medical areas.

^c^ includes community specialty clinic and palliative care.

^d^ Multiple options could be selected.

^e^ Measured on 7‐point Likert scale; 1 = very unconfident, 4 = neutral, 7 = very confident.

Compared with metropolitan participants, significantly fewer rural participants were exposed to suspected (rural = 46.3%; metropolitan = 57.9%) or confirmed (rural = 7.1%; metropolitan = 29.6%) COVID‐19 patients or worked with patients with COVID‐19 (rural = 22.3%; metropolitan = 42.9%) (Table 3). Fewer rural participants had received training to care for COVID‐19 patients (rural = 26.7%; metropolitan = 37.6%) or use PPE (rural = 56.4%; metropolitan = 67.6%). Rural participants were overall less confident with caring for COVID‐19 patients (rural = 1.32; metropolitan = 1.50) and using COVID‐19 PPE (rural = 1.59; metropolitan = 1.70) and were more likely to desire additional training compared to metropolitan HCWs (rural = 57.8%; metropolitan = 48.9%) (Table 3).

**TABLE 3:** Experiences with COVID‐19

	Rural *n* = 1473 *n* (%)	Metropolitan *n* = 6373 *n* (%)	*p*‐value
Currently working with people infected with COVID‐19	329 (22.3)	2734 (42.9)	<0.001
Anticipates working with people infected with COVID‐19	683 (60.1)	2208 (60.7)	0.708
Exposure to confirmed COVID‐19 patients	104 (7.1)	1888 (29.6)	<0.001
Exposure to suspected COVID‐19 patients	679 (46.3)	3686 (57.9)	<0.001
Received training to care for patients with COVID‐19	393 (26.7)	2399 (37.6)	<0.001
Confidence caring for people with COVID‐19 (mean [SD]) ^a^	1.32 (0.82)	1.50 (0.76)	<0.001
Received training on PPE during the pandemic	830 (56.4)	4307 (67.6)	<0.001
Confidence using PPE (mean [SD])^a^	1.59 (0.69)	1.70 (0.61)	<0.001
Desires more training regarding PPE or managing people with COVID‐19	585 (57.8)	2416 (48.9)	<0.001
Worried about being blamed by colleagues if they contract COVID‐19			
Neither agree/disagree	263 (17.9)	1012 (15.9)	0.002
Disagree	258 (17.5)	1364 (21.4)	
Agree	952 (64.6)	3997 (62.7)	
Worried their role will lead to transmission of COVID‐19 to family			
Not worried	139 (13.7)	590 (11.9)	0.279
Neutral	111 (11.0)	563 (11.4)	
Very worried	762 (75.3)	3789 (76.7)	
Quarantined or furloughed due to exposure to COVID‐19	74 (5.0)	703 (11.0)	<0.001
Undergone testing for COVID‐19	1048 (71.2)	5110 (80.2)	<0.001
Tested positive for COVID‐19	2 (0.2)	134 (2.6)	<0.001
Close friends/relatives infected with COVID‐19 in Australia or overseas	263 (17.9)	2135 (33.5)	<0.001

Abbreviation: PPE, personal protective equipment.

^a^ Measured on 7‐point Likert scale; 1 = very unconfident, 4 = neutral, 7 = very confident.

### 3.2 Mental health

Prior to the pandemic, significantly more rural participants had a mental health issue compared to metropolitan participants (rural = 35.0%; metropolitan = 29.4%; *p* < 0.001). There were no significant differences between the two groups in relation to prevalence of self‐determined mental health issues experienced during COVID‐19. Both groups reported similarly high levels of resilience (rural = 3.24; metropolitan = 3.21).

However, there were differences between the prevalence of objectively measured mental health outcomes reported by rural and metropolitan participants. Rural participants had a significantly higher prevalence of high emotional exhaustion (rural = 46.5%; metropolitan = 43.3%; *p* = 0.002). Metropolitan participants experienced significantly higher rates moderate to severe PTSD (rural = 41.0%; metropolitan = 38.0; *p* = 0.031), depersonalisation (rural = 20.7%; metropolitan = 18.1%; *p* = 0.047) and moderate personal accomplishment (rural = 21.2%; metropolitan = 18.6%; *p* = 0.035). Less than 30% of participants from both groups sought help for stress or mental health problems from a mental health practitioner or from a workplace or external support program. (Table 4).

**TABLE 4:** Prevalence of mental health symptoms

	Rural *n* = 1473 *n* (%)	Metropolitan *n* = 6373 *n* (%)	*p*‐value
Previous mental health condition (Yes)	515 (35.0)	1874 (29.4)	<0.001
Subjective self‐reported mental health problems experienced since COVID‐19 pandemic commenced			
Anxiety	887 (60.2)	3988 (62.6)	0.092
Depression	417 (28.3)	1758 (27.6)	0.576
Post‐traumatic stress disorder	78 (5.3)	349 (5.5)	0.783
Burnout	840 (57.0)	3735 (58.6)	0.268
Other mental health issue	63 (4.3)	265 (4.2)	0.837
No mental health issues	279 (18.9)	1152 (18.1)	0.439
Objective mental health symptoms assessed by validated scales			
Anxiety (GAD7)	*n* = 1473	*n* = 6370	
None—mild	1047 (71.1)	4579 (71.9)	0.537
Moderate—severe	426 (28.9)	1791 (28.1)	
Depression (PHQ9)	*n* = 1463	*n* = 6353	
None—mild	1110 (75.9)	4883 (76.9)	0.420
Moderate—severe	353 (24.1)	1470 (23.1)	
Impact of events/trauma (IES‐6)	*n* = 1459	*n* = 6337	
None—mild	905 (62.0)	3736 (59.0)	0.031
Moderate–severe	554 (38.0)	2601 (41.0)	
Burnout—depersonalisation (DP)	*n* = 1436	*n* = 6252	0.047
Low	936 (65.2)	3875 (62.0)	
Moderate	240 (16.7)	1081 (17.3)	
High	260 (18.1)	1296 (20.7)	
Burnout—emotional exhaustion (EE)	*n* = 1440	*n* = 6261	0.002
Low	436 (30.3)	1807 (28.9)	
Moderate	335 (23.3)	1744 (27.9)	
High	669 (46.5)	2710 (43.3)	
Burnout—personal accomplishment (PA)	*n* = 1438	*n* = 6251	0.035
Low	433 (30.1)	1925 (30.8)	
Moderate	267 (18.6)	1325 (21.2)	
High	738 (51.3)	3001 (48.0)	
	Mean (SD)	Mean (SD)	
Resilience CD‐RISC‐2	3.24 (0.642)	3.21 (0.665)	0.132
Mental health help‐seeking behaviours			
Doctor or psychologist	285 (19.3)	1151 (18.1)	0.249
Employee support program through work	102 (6.9)	372 (5.8)	0.114
Support program external to work	58 (3.9)	118 (2.9)	0.050
Other	54 (3.7)	256 (4.0)	0.533
None	1056 (71.7	4737 (74.3)	0.038

GAD7: 0–4 = none/minimal, 5–9 = mild, 10–14 = moderate, 15–21 = severe anxiety; PHQ9: 0–4 = none/minimal, 5–9 = mild, 10–14 = moderate, 15–19 = moderately severe, and 20–27 = severe; IES‐6 is categorised as 0–9 = none‐mild, >9 = moderate–severe.

Burnout is indicated by higher scores on the EE and DP, and lower scores on PA; Burnout DP: 0–3 = low, 4–6 = moderate, 7–18 = high; Burnout EE: 0–6 = low, 7–10 = moderate, 11–18 = high; Burnout PA: 15–18 = low, 13–14 = moderate, 0–13 = high.

CD‐RISC‐2 scale: 2 items, each rated on a 5‐point scale (0–4), higher scores reflecting greater resilience.

### 3.3 Factors associated with mental health outcomes

**FIGURE 1:**
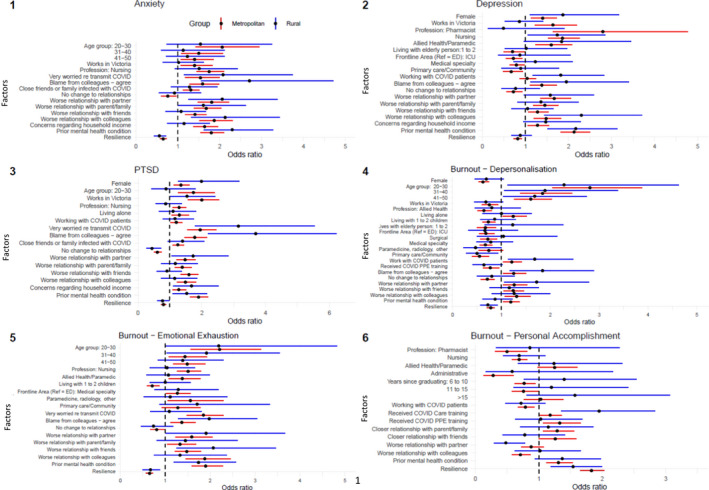
Personal and workplace predictors of mental health symptoms in rural and metropolitan participants. Predictors for rural participants are indicated in blue and metropolitan participants in red. Only significant associations are shown. Bars are indicative of odds ratio (OR) with 95% confidence intervals for experiencing symptoms (on validated scales) of: 1) anxiety, 2) depression, 3) post‐traumatic stress disorder (PTSD) and burnout subdomains of 4) depersonalisation, 5) emotional exhaustion and 6) personal accomplishment (PA). For all plots (except PA), OR <1 indicate the covariate is protective, OR >1 indicate the covariate is a predictor. Lower odds ratio for personal accomplishment indicates poorer outcomes. Reference categories: worse relationship with friends, family, partner or colleagues vs no change, prior mental illness vs none, income concerns vs none, concerns of blame by colleagues vs negative response, concerns of infecting family vs negative response, female vs male, Victoria vs other states, frontline areas (ICU, medical specialty, primary care, anaesthetics and surgical) vs emergency department, PPE training vs none, age (ordinal variable <20 years), lives alone vs with others, lives with children vs does not

For both rural and metropolitan participants, higher levels of anxiety were independently associated with being worried about transmitting COVID‐19 to family (rural OR = 2.08, [95%CI 1.15–3.75]; metropolitan OR = 1.54, [1.18–2.00]), being concerned about being blamed by colleagues if they contracted COVID‐19 (rural OR = 2.71, [1.56–4.71]; metropolitan OR = 1.59, [1.28–1.98]) and the pandemic adversely impacting their relationships with partner (rural OR = 2.06, [1.26–3.38]; metropolitan OR = 1.81, [1.47–2.22]) or colleagues (rural OR = 2.13, [1.32–3.44]; metropolitan OR = 1.87, [1.52–2.31]). Participants were less likely to experience higher anxiety levels if they were resilient in both rural (OR = 0.56, [0.43–0.72]) and metropolitan settings (OR = 0.65, [0.58–0.73]). (Figure 1, Table S1).

Higher levels of depression, for both locations, were independently associated with being female (rural OR = 1.87, [1.10–3.18]; metropolitan OR = 1.40, [1.13–1.73]), being a nurse (rural OR = 1.74, [1.06–2.86]; metropolitan OR = 1.86, [1.53–2.26]), being worried about being blamed by colleagues if they contracted COVID‐19 (rural OR = 1.95, [1.12–3.40]; metropolitan OR = 1.38, [1.10–1.73]), the pandemic adversely impacting their relationships with colleagues (rural OR = 2.30, [1.43–3.71]; metropolitan OR = 1.48, [1.19–1.83]) and having a prior mental health condition (rural OR = 2.17, [1.51–3.14[; metropolitan OR = 2.13, [1.82–2.50]). In rural areas, the only significant association was working with COVID‐19 patients (OR = 1.82, [1.17–2.83]). (Figure 1, Table S2).

Higher levels of PTSD for both groups were independently associated with being female (rural OR = 1.99, [1.25–3.18]; metropolitan OR = 1.35, [1.12–1.61]), being worried about transmitting COVID‐19 to family (rural OR = 3.15, [1.79–5.54]; metropolitan OR = 1.95, [1.55–2.45]), being blamed by colleagues if they contracted COVID‐19 (rural OR = 3.69, [2.20–6.22]; metropolitan OR = 1.78, [1.46–2.17]), the pandemic adversely impacting their relationship with a partner (rural OR = 1.73, [1.06–2.84]; metropolitan OR = 1.48, [1.22–1.83]), having concerns about finances (rural OR = 1.68, [1.11–2.53]; metropolitan OR = 1.28, [1.08–1.52]) and having a prior mental health condition (rural OR = 1.53, [1.08–2.16]; metropolitan OR = 1.90, [1.64–2.20]). Lower levels of PTSD for both groups were associated with experiencing no change to personal or work relationships (rural OR = 0.44, [0.27–0.72]; metropolitan OR = 0.80, [0.72–0.88]) and reporting higher levels of resilience (rural OR = 0.77, [0.60–0.99]; metropolitan OR = 0.80, [0.72–0.88]) (Figure 1, Table S3).

Multiple factors were independently associated with higher levels of depersonalisation (subdomain of burnout) for both groups including younger age (rural OR = 2.28, [1.13–4.63]; metropolitan OR = 2.81, [2.04–3.88]), working with COVID‐19 patients (rural OR = 1.68, [1.14–2.47]; metropolitan OR = 1.21, [1.04–1.42]), having concerns about being blamed by colleagues (rural OR = 1.084, [1.17–2.89]; metropolitan OR = 1.25, [1.03–1.51]) and the pandemic adversely impacting their relationship with a partner (rural OR = 1.72, [1.06–2.79]; metropolitan OR = 1.26, [1.03–1.53]). Protective factors included working in primary health care or community care (rural OR = 0.50, [0.27–0.93]; metropolitan OR = 0.55, [0.40–0.74]) or having higher levels of resilience (rural OR = 0.72, [0.56–0.92]; metropolitan OR = 0.78, [0.70–0.86]) (Figure 1, Table S4).

Factors independently associated with higher levels of emotional exhaustion (subdomain of burnout) for both groups included being worried about being blamed by colleagues if they contracted COVID‐19 (rural OR = 1.98, [1.28–3.05]; metropolitan OR = 1.37, [1.12–1.67]), the pandemic adversely impacting their relationship with friends (rural OR = 2.07, [1.25–3.46]; metropolitan OR = 1.48, [1.45–2.45]) or having a prior mental health condition (rural OR = 1.74, [1.19–2.57]; metropolitan OR = 1.90, [1.59–2.28]). Having higher resilience was a protective factor for both groups (rural OR = 0.67, [0.50–0.89]; metropolitan OR = 0.65, [0.57–0.73]) (Figure 1, Table S5).

The factor associated with lower levels of personal accomplishment (subdomain of burnout) among the rural group was the pandemic adversely impacting their relationship with a partner (OR = 0.48, [0.29–0.78]). The factors associated with higher levels of personal accomplishment among both groups included receiving COVID‐19 care training (rural OR = 1.95, [1.35–2.83]; metropolitan OR = 1.18, [1.02–1.36]) and having higher levels of resilience (rural OR = 1.54, [1.19–2.00]; metropolitan OR = 1.83, [1.65–2.03]). (Figure 1, Table S6).

## 4 Discussion

To our knowledge, this is the largest, national, cross‐sectional survey that has included a breadth of HCWs with people working across the primary, secondary and tertiary care sectors from rural and metropolitan Australia. Of note, it is the largest study examining the psychosocial impacts of COVID‐19 pandemic on rural HCWs. Concerningly, despite this being a period of low COVID‐19 prevalence nationally and extremely low in rural areas, approximately 82% of participants self‐reported having experienced mental health concerns during this period of the COVID‐19 pandemic, as well as moderate to high levels of self‐reported anxiety, burnout and depression. These self‐reported experiences were consistent with the objective measures using the psychological assessment tools. A high prevalence of moderate–severe anxiety, burnout and depression, and even higher prevalence of moderate to severe PTSD were documented. These findings are consistent with the international literature.^28^ These mental health effects are profound and are indicative of the levels of disruption and distress that the participants experienced during the pandemic.

Multiple factors might have contributed to HCWs' psychosocial distress including fear associated with the speed at which COVID‐19 was spreading across the world,^29^ early limited understanding of the SARS‐COV2 virus and its implications,^9^ not knowing when the crisis would end,^7^ altered clinical practices, with adoption of infection control measures beyond usual practice,^13^, ^30^ increased long‐term workload,^5^ shortage of adequate medical and infection control equipment,^9^ staff shortages associated with frequent staff furloughs, being isolated from family, friends and colleagues,^31^ inadequate rest and recuperation time,^9^ and contemplating and/or confronting critical life events such as severe morbidity and death.^29^ This study identified multiple workplace and personal factors associated with mental health effects, which have also been observed in other studies: worry about contracting and transmitting COVID‐19 to others,^8^ concerned about being blamed by other colleagues if they contract COVID‐19,^7^ need for additional clinical training,^32^ COVID‐19 having adverse impacts on their personal and professional relationships or having a mental health condition prior to COVID‐19 pandemic.^31^ Although less than a quarter of rural participants worked with COVID‐19 patients during this period, their high levels of concern about being blamed by their colleagues if they contracted the disease or concern about transmitting the virus to others in their household were still significantly associated with anxiety, depression and PTSD. Protective factors were also identified: having higher levels of resilience ^33^ and receiving training and support for managing COVID‐19. ^6^ Despite a very low COVID‐19 caseload in rural Australia during the study, workplace changes within rural health services were extensive. This included increased hours (paid or unpaid), changes in income and role changes. Such factors might worsen worry, stress or anxiety irrespective of caseload.^31^

These findings outline the multiple adverse and protective factors that should be considered when planning flexible and strategic interventions to manage or minimise psychosocial distress across the full range of health care workers. These findings are consistent with other studies that have focussed on HCWs in metropolitan areas. ^9^, ^31^, ^34^ As such, this study provides an evidence‐based platform to guide the development of policies and procedures for planning into the future to reduce the mental health impact on HCWs in both rural and metropolitan regions.

Adverse impacts on mental health were equally prevalent among the rural and metropolitan participants, and rural participants had higher levels of burnout. These findings differed from a cross‐sectional study of HCWs and non‐HCWs in China during February to March 2020, which reported that rurally based HCWs were at higher risk of anxiety, depression, insomnia and obsessive–compulsive symptoms.^8^ The authors explained that this higher risk might have been associated with rural HCWs being more worried about being infected due to changed medical conditions and requirements for different clinical skills. This difference might be due to the prevalence and severity of the COVID‐19 epidemic in China during that period and the higher density of rural dwelling people in the region investigated.

Given the multiple adverse impacts on mental health, it is concerning that reported help‐seeking behaviours were low. The participants were well aware of the mental health impact and their self‐reports mirrored the objective measures. Prior to COVID‐19 pandemic, a high prevalence of anxiety, depression and burnout had been documented among HCWs ^35^, ^36^. Similarly, a high prevalence of pre‐existing mental health conditions was reported in this study sample and was significantly higher among the rural group. Rural HCWs face challenges that are not experienced in metropolitan settings such as less access to specialist care, difficulties in recruitment and retention, less availability of locum HCWs, infrastructure challenges, limited access to continuing professional development and fewer support services.^18^ Most psychological help during this period of COVID‐19 pandemic was sourced through psychologists or doctors rather than through other support programs. Further investigation is required to understand how help was sought for mental health issues. Barriers to access to health care for rural HCWs has been documented,^37^, ^38^, ^39^ even before the pandemic, and clearly needs to be addressed effectively into the future.

Only about half of rural participants received training for managing COVID‐19 patients and PPE and this represented 10% less than their metropolitan counterparts, which highlights a need for training. Training in COVID‐19 care and using PPE was related to higher levels of personal accomplishment (and therefore lower risk of burnout). Hence, gaps in training are likely to impact on workforce mental health and well‐being. Similar results were reported from the UK during the early stage of the COVID‐19 pandemic.^32^ As the COVID‐19 pandemic continues into a third year with rising incidence of new cases across Australia,^40^ particularly among the most vulnerable and at‐risk communities, it is crucial that surge capacity in health services is assured. Gaps in training need to be bridged, improving knowledge, practices, responsiveness and confidence of HCWs. In addition to providing appropriate health care to COVID‐19 patients, this should be included as one aspect of the planning to enhance the mental health and well‐being of HCWs, thereby supporting a sustainable rural health workforce.^41^, ^42^

### 4.1 Implications

Proactive and targeted training strategies are needed to support and enable rural health care workforce immediately and ongoing as the COVID‐19 pandemic evolves. The large internal migration of people from metropolitan to rural areas during the COVID‐19 pandemic inevitably puts pressure on already stressed rural health care services. In rural Australia, for all health professions, the number of employed clinicians per capita serviced working in their registered professions decreases with increasing remoteness from metropolitan areas.^43^ As the metropolitan health care workforce are required for the increased workload associated with COVID‐19 in cities, it might not be possible to redeploy metropolitan staff to regional and rural areas readily. This highlights the importance of future planning to increase the health workforce capacity.

### 4.2 Strengths and Limitations

The large sample size across metropolitan and rural areas of Australia enabled examination of independent factors of mental health status. The majority of participants were women (80.2%), and this is consistent with Australian health workforce demographic data,^43^ which reports that the ratio of women is approximately 2.5 times that of men (75%).

The psychometric instruments used have been validated across the general population and specific subpopulations, including HCWs. They have been utilised extensively throughout research that has examined the impact of COVID‐19 pandemic on the general population and HCWs in numerous settings, which will enable comparison between studies and provide a baseline of mental health impacts.

Due to the very broad survey dissemination and recruitment strategy, we do not know the size of the population invited to participate; hence, we cannot calculate the response rate. As the sampling strategy was a non‐probability‐based sampling method and the source population and the sampling frame is unknown, it is possible that selection bias might have resulted in and under‐ or over‐estimation of pre‐existing mental health conditions or experiences of psychological distress in this self‐selected study sample. Also, as a result the unweighted prevalence and effect estimates reported in this study are likely to be different to the true estimates in the population. Misclassification bias might also affect these findings as clinical assessment of mental health outcomes to verify the symptoms measured by the validated psychological scales was not possible due to the nature of the study being a survey of large sample size during a pandemic.

The cross‐sectional design of this study limits understanding as to how the identified factors associated with the mental health outcomes could impact in the longer‐term. Within this context, it is important to understand that the factors identified are associated with the outcomes based on clinical plausibility and statistical significance, thus causality cannot be demonstrated. However, these findings add to the emerging global body of knowledge about the risk factors for mental health impacts on frontline workers in health care settings during a pandemic.

Participant responses were obtained at a single time point rather than at multiple time points to avoid burdening HCWs during this COVID‐19 pandemic. At the time of this survey, rural Australia had not been significantly impacted by the direct spread of COVID‐19 and its associated symptoms and effects through the communities. The prolonged nature of the pandemic, including the emergence of multiple variants of concern of SARS‐CoV2 virus, such as Omicron BA.1 and BA.2, which have spread rapidly through areas previously untouched by COVID‐19 and have placed the health system under extreme pressure,^44^, ^45^ requires longitudinal research to be undertaken to ensure better understanding of emerging or persisting psychosocial effects and implications for patient, HCW and community safety, and workforce support, training and retention.

## 5 Conclusion

The rural health workforce is an indispensable, highly trained and committed workforce that needs appropriate support and protection to ensure the delivery of high‐quality and safe health care services for regional, rural and remote Australia are maintained. Health care services throughout rural Australia need to be aware of the widespread mental health impacts of COVID‐19 within their health care workforce. This study supports the need for ongoing evidence‐based policies and practices to ensure supportive mechanisms and adequate mental health services are in place for prevention, early detection and effective management for adverse psychological effects.

## CONFLICT OF INTEREST

The authors declare no conflict of interest.

## AUTHOR CONTRIBUTIONS

NS: conceptualization; formal analysis; funding acquisition; methodology; project administration; supervision; writing – original draft. MK: writing – review and editing. KW: conceptualization; funding acquisition; methodology; project administration; writing – review and editing. AP: data curation; project administration; writing – review and editing. RT: formal analysis; writing – original draft.

## ETHICAL APPROVAL

Ethics approval was provided by the Royal Melbourne Hospital Human Research Ethics Committee (HREC/67074/MH‐2020).

## INFORMED CONSENT STATEMENT

Informed consent was obtained from all subjects involved in the study.

## Supporting information


Table S1‐S6
