# Supplementary material for: Differences in psychosocial distress among rural and metropolitan health care workers during the COVID‐19 pandemic
Source: Aust J Rural Health. 2022 May 5;30(5):683–96. doi: 10.1111/ajr.12873 (PMC9347496; doi:10.1111/ajr.12873)
Supplement: Supplementary file 1 — Table S1‐S6 [file AJR-30-683-s001.docx]

### Supplementary Table 1: Multivariate logistic regression of anxiety factors by rural and metropolitan locations

| **Anxiety (GAD-7)** | **Rural** |  | **Metropolitan** |  |
| --- | --- | --- | --- | --- |
|  | **OR (95%CI)** | **p** | **OR (95%CI)** | **p** |
| **PERSONAL FACTORS** |  |  |  |  |
| Female | 1.20 (0.74-1.95) | 0.450 | 1.19 (0.97-1.45) | 0.097 |
| Age group (Ref 50+) |  |  |  |  |
| 20-30 | 1.54 (0.73-3.25) | 0.262 | 2.06 (1.43-2.95) | <0.001 |
| 31-40 | 1.13 (0.60-2.11) | 0.711 | 1.50 (1.08-2.08) | 0.015 |
| 41-50 | 1.23 (0.72-2.11) | 0.451 | 1.40 (1.07-1.85) | 0.016 |
| State (Ref = Others) |  |  |  |  |
| Victoria | 1.02 (0.64-1.62) | 0.943 | 1.40 (1.07-1.85) | 0.016 |
| Health role (ref = Medical practitioner) |  |  |  |  |
| Pharmacist | 1.83 (0.70-4.76) | 0.216 | 1.70 (0.99-2.90) | 0.054 |
| Nursing | 1.51 (0.93-2.43) | 0.094 | 1.75 (1.45-2.10) | <0.001 |
| Allied Health/Paramedic/ Clinical Scientist | 1.28 (0.69-2.36) | 0.436 | 1.26 (0.98-1.63) | 0.072 |
| Other/Support/Leader/Clerical/Administrative | 1.29 (0.29-5.81) | 0.738 | 1.58 (0.65-3.83) | 0.309 |
| Years of experience since graduating (Ref = 0-5) |  |  |  |  |
| 6 to 10 | 1.69 (0.94-3.05) | 0.080 | 0.90 (0.72-1.12) | 0.334 |
| 11 to 15 | 1.12 (0.54-2.32) | 0.756 | 0.77 (0.57-1.03) | 0.076 |
| >15 | 1.18 (0.59-2.34) | 0.639 | 0.95 (0.69-1.30) | 0.729 |
| Living alone (Ref = No) | 0.83 (0.48-1.42) | 0.080 | 1.16 (0.93-1.45) | 0.194 |
| Living with children (ref = 0) |  |  |  |  |
| 1 to 2 | 0.92 (0.59-1.44) | 0.704 | 0.89 (0.72-1.09) | 0.244 |
| 3 to 4 | 1.10 (0.53-2.29) | 0.793 | 1.04 (0.75-1.45) | 0.816 |
| Living with elderly (Ref = 0) |  |  |  |  |
| 1 to 2 | 1.10 (0.57-2.10) | 0.778 | 1.01 (0.74-1.36) | 0.972 |
| 3+ | - | - | 2.17 (0.86-5.46) | 0.100 |
| Changed household incomes (Ref = Increased) |  |  |  |  |
| Decreased | 0.81 (0.42-1.58) | 0.543 | 0.82 (0.62-1.08) | 0.163 |
| No change | 0.94 (0.55-1.60) | 0.812 | 0.87 (0.69-1.11) | 0.266 |
| Concerns regarding household income (Ref = No) | 1.15 (0.75-1.76) | 0.531 | 1.64 (1.37-1.96) | <0.001 |
| Close friends or family infected with Covid (Ref = No) | 1.28 (0.84-1.95) | 0.244 | 1.31 (1.12-1.52) | <0.001 |
| Prior mental health condition (Ref = No/NA) | 2.30 (1.62-3.28) | <0.001 | 1.80 (1.54-2.10) | <0.001 |
| Resilience | 0.56 (0.43-0.72) | <0.001 | 0.65 (0.58-0.73) | <0.001 |
| No change to relationships with family or friends or colleagues (ref = No) | 0.92 (0.55-1.56) | 0.766 | 0.76 (0.60-0.97) | 0.025 |
| Closer relationship with parent/family (Ref = No) | 1.21 (0.75-1.96) | 0.429 | 0.98 (0.81-1.20) | 0.875 |
| Closer relationship with friends (Ref = No) | 1.00 (0.54-1.86) | 0.995 | 0.94 (0.74-1.19) | 0.596 |
| Worse relationship with partner (Ref = No) | 2.06 (1.26-3.38) | 0.004 | 1.81 (1.47-2.22) | <0.001 |
| Worse relationship with parent/family (Ref = No) | 1.61 (0.99-2.62) | 0.058 | 1.68 (1.38-2.04) | <0.001 |
| Worse relationship with friends (Ref = No) | 1.08 (0.70-1.67) | 0.723 | 1.41 (1.18-1.69) | <0.001 |
| Worse relationship with colleagues (Ref = No) | 2.13 (1.32-3.44) | 0.002 | 1.87 (1.52-2.31) | <0.001 |
| **WORKPLACE FACTORS** |  |  |  |  |
| Frontline category (Ref = ED) |  |  |  |  |
| ICU | 1.04 (0.45-2.39) | 0.933 | 0.89 (0.69-1.15) | 0.367 |
| Anaesthetics/Periop/Surgical | 1.89 (0.86-4.14) | 0.113 | 1.14 (0.87-1.49) | 0.343 |
| Medical specialty areas with general medicine, Hospital aged care | 1.19 (0.70-2.01) | 0.524 | 1.13 (0.91-1.40) | 0.256 |
| Other (Paramedicine, radiology, pathology, maintenance, clerical, admin,) | 1.55 (0.71-3.35) | 0.268 | 1.11 (0.77-1.61) | 0.565 |
| Primary care/Community, Residential or non-hospital aged care | 1.45 (0.75-2.82) | 0.273 | 1.00 (0.72-1.39) | 0.993 |
| Working with COVID patients (Ref = No) | 1.38 (0.90-2.12) | 0.140 | 1.12 (0.95-1.33) | 0.187 |
| Received COVID Care training (Ref = No) | 0.78 (0.54-1.13) | 0.187 | 0.98 (0.84-1.14) | 0.815 |
| Received COVID PPE training (Ref = No) | 0.69 (0.42-1.13) | 0.140 | 0.87 (0.69-1.11) | 0.261 |
| Worried re transmitting Covid to family (ref = Not worried) |  |  |  |  |
| Neutral | 0.96 (0.43-2.16) | 0.921 | 0.89 (0.63-1.25) | 0.505 |
| Very worried | 2.08 (1.15-3.75) | 0.015 | 1.54 (1.18-2.00) | 0.001 |
| Worried about being blamed by colleagues (Ref = Neutral) |  |  |  |  |
| Strongly/somewhat disagree | 2.09 (1.07-4.09) | 0.031 | 0.089 (0.68-1.16) | 0.385 |
| Strongly/somewhat agree | 2.71 (1.56-4.71) | <0.001 | 1.59 (1.28-1.98) | <0.001 |

### Supplementary Table 2: Multivariate logistic regression of depression factors by rural and metropolitan locations

| **Depression – PHQ9** | **Rural** |  | **Metropolitan** |  |
| --- | --- | --- | --- | --- |
|  | **OR (95%CI)** | **p** | **OR (95%CI)** | **p** |
| **PERSONAL FACTORS** |  |  |  |  |
| Female | 1.87 (1.10-3.18) | 0.022 | 1.40 (1.13-1.73) | 0.002 |
| Age group (Ref 50+) |  |  |  |  |
| 20-30 | 1.18 (0.55-2.53) | 0.662 | 1.32 (0.91-1.92) | 0.137 |
| 31-40 | 0.91 (0.48-1.73) | 0.78 | 1.08 (0.78-1.51) | 0.638 |
| 41-50 | 1.21 (0.70-2.10) | 0.489 | 1.23 (0.93-1.63) | 0.142 |
| State (Ref = Others) |  |  |  |  |
| Victoria | 0.86 (0.52-1.41) | 0.545 | 1.64 (1.22-2.20) | 0.001 |
| Profession (ref = Medical practitioner) |  |  |  |  |
| Pharmacist | 0.49 (0.13-1.90) | 0.305 | 2.80 (1.64-4.78) | <0.001 |
| Nursing | 1.74 (1.06-2.86) | 0.030 | 1.86 (1.53-2.26) | <0.001 |
| Allied Health/Paramedic/ Clinical Scientist | 1.84 (0.97-3.46) | 0.061 | 1.60 (1.23-2.08) | <0.001 |
| Other/Support/Leader/Clerical/Administrative | 2.59 (0.61-10.91) | 0.195 | 0.68 (0.22-2.10) | 0.499 |
| Years of experience since graduating (Ref = 0-5) |  |  |  |  |
| 6 to 10 | 1.59 (0.87-2.93) | 0.133 | 0.91 (0.73-1.14) | 0.400 |
| 11 to 15 | 1.31 (0.62-2.77) | 0.474 | 0.78 (0.58-1.06) | 0.114 |
| >15 | 1.03 (0.51-2.08) | 0.939 | 0.89 (0.64-1.23) | 0.466 |
| Living alone (Ref = No) | 0.91 (0.52-1.59) | 0.746 | 1.20 (0.95-1.50) | 0.121 |
| Living with children (ref = 0) |  |  |  |  |
| 1 to 2 | 1.24 (0.79-1.97) | 0.350 | 0.82 (0.66-1.01) | 0.062 |
| 3 to 4 | 1.41 (0.67 – 2.97) | 0.361 | 1.04 (0.74-1.47) | 0.818 |
| Living with elderly (Ref = 0) |  |  |  |  |
| 1 to 2 | 1.02 (0.52-1.97) | 0.961 | 0.70 (0.50-0.98) | 0.039 |
| 3+ | --- | - | 0.91 (0.34-2.41) | 0.844 |
| Changed household incomes (Ref = Increased) |  |  |  |  |
| Decreased | 0.94 (0.46-1.90) | 0.862 | 0.89 (0.67-1.19) | 0.435 |
| No change | 1.33 (0.76-2.35) | 0.319 | 0.87 (0.68-1.11) | 0.267 |
| Concerns regarding household income (Ref = No) | 1.47 (0.95-2.29) | 0.087 | 1.28 (1.06-1.55) | 0.010 |
| Close friends or family infected with Covid (Ref = No) | 0.99 (0.64-1.54) | 0.958 | 1.05 (0.89-1.22) | 0.575 |
| Prior mental health condition (Ref = No/NA) | 2.17 (1.51-3.14) | <0.001 | 2.13 (1.82-2.50) | <0.001 |
| Resilience | 0.88 (0.67-1.15) | 0.345 | 0.82 (0.73-0.91) | <0.001 |
| No change to relationships with family or friends or colleagues (ref = No) | 0.77 (0.44-1.33) | 0.348 | 0.72 (0.56-0.93) | 0.010 |
| Closer relationship with parent/family (Ref = No) | 1.45 (0.89-2.36) | 0.141 | 0.93 (0.76-1.14) | 0.505 |
| Closer relationship with friends (Ref = No) | 1.11 (0.60-2.04) | 0.746 | 0.98 (0.77-1.25) | 0.871 |
| Worse relationship with partner (Ref = No) | 1.58 (0.96-2.59) | 0.071 | 1.67 (1.35-2.06) | <0.001 |
| Worse relationship with parent/family (Ref = No) | 1.36 (0.82-2.24) | 0.233 | 1.45 (1.18-1.77) | <0.001 |
| Worse relationship with friends (Ref = No) | 1.05 (0.67-1.65) | 0.819 | 1.28 (1.07-1.54) | 0.008 |
| Worse relationship with colleagues (Ref = No) | 2.30 (1.43-3.71) | 0.001 | 1.48 (1.19-1.83) | <0.001 |
| **WORKPLACE FACTORS** |  |  |  |  |
| Frontline category (Ref = ED) |  |  |  |  |
| ICU | 0.87 (0.37-2.05) | 0.757 | 0.72 (0.55-0.93) | 0.013 |
| Anaesthetics/Periop/Surgical | 0.99 (0.43-2.28) | 0.983 | 0.89 (0.68-1.17) | 0.407 |
| Medical specialty areas with general medicine, Hospital aged care | 1.23 (0.73-2.09) | 0.436 | 0.79 (0.63-0.98) | 0.032 |
| Other (Paramedicine, radiology, pathology, maintenance, clerical, admin) | 0.73 (0.32-1.69) | 0.465 | 0.74 (0.51-1.07) | 0.110 |
| Primary care/Community, Residential or non-hospital aged care | 0.89 (0.45-1.78) | 0.746 | 0.67 (0.48-0.94) | 0.021 |
| Working with COVID patients (Ref = No) | 1.82 (1.17-2.83) | 0.008 | 1.04 (0.87-1.24) | 0.694 |
| Received COVID Care training (Ref = No) | 0.78 (0.53-1.14) | 0.198 | 0.97 (0.82-1.13) | 0.662 |
| Received COVID PPE training (Ref = No) | 0.78 (0.46-1.32) | 0.364 | 0.89 (0.70-1.15) | 0.374 |
| Worried re transmitting Covid to family (ref = Not worried) |  |  |  |  |
| Neutral | 0.77 (0.35-1.66) | 0.503 | 1.02 (0.73-1.42) | 0.899 |
| Very worried | 1.00 (0.57-1.75) | 1.00 | 1.16 (0.90-1.51) | 0.251 |
| Worried about being blamed by colleagues (Ref = Neutral) |  |  |  |  |
| Strongly/somewhat disagree | 1.36 (0.68-2.72) | 0.388 | 0.91 (0.69-1.20) | 0.515 |
| Strongly/somewhat agree | 1.95 (1.12-3.40) | 0.018 | 1.38 (1.10-1.73) | 0.006 |

### Supplementary Table 3: Multivariate logistic regression of post-traumatic stress disorder factors by rural and metropolitan locations

| **Post-Traumatic Stress Disorder – IES-6** | **Rural** |  | **Metropolitan** |  |
| --- | --- | --- | --- | --- |
|  | **OR (95%CI)** | **p** | **OR (95%CI)** | **p** |
| **PERSONAL FACTORS** |  |  |  |  |
| Female | 1.99 (1.25-3.18) | 0.004 | 1.35 (1.12-1.61) | 0.001 |
| Age group (Ref 50+) |  |  |  |  |
| 20-30 | 0.88 (0.42-1.81) | 0.720 | 1.74 (1.25-2.41) | 0.001 |
| 31-40 | 1.22 (0.67-2.23) | 0.509 | 1.28 (0.96-1.71) | 0.096 |
| 41-50 | 1.36 (0.81-2.28) | 0.245 | 1.06 (0.84-1.35) | 0.608 |
| State (Ref = Others) |  |  |  |  |
| Victoria | 1.54 (0.98-2.42) | 0.061 | 2.00 (1.57-2.55) | <0.001 |
| Profession (ref = Medical practitioner) |  |  |  |  |
| Pharmacist | 1.27 (0.50-3.21) | 0.618 | 1.47 (0.88-2.45) | 0.138 |
| Nursing | 0.87 (0.56-1.37) | 0.560 | 1.29 (1.09-1.52) | 0.003 |
| Allied Health/Paramedic/ Clinical Scientist | 1.28 (0.71-2.28) | 0.410 | 1.18 (0.94-1.49) | 0.149 |
| Other/Support/Leader/Clerical/Administrative | 3.92 (0.99-15.51) | 0.052 | 0.88 (0.38-2.08) | 0.776 |
| Years of experience since graduating (Ref = 0-5) |  |  |  |  |
| 6 to 10 | 0.74 (0.41-1.31) | 0.301 | 1.06 (0.86-1.30) | 0.587 |
| 11 to 15 | 0.90 (0.45-1.79) | 0.756 | 0.83 (0.63-1.08) | 0.168 |
| >15 | 0.72 (0.37-1.39) | 0.322 | 0.98 (0.74-1.32) | 0.913 |
| Living alone (Ref = No) | 1.10 (0.66-1.82) | 0.713 | 1.30 (1.05-1.60) | 0.014 |
| Living with children (ref = 0) |  |  |  |  |
| 1 to 2 | 0.85 (0.55-1.30) | 0.442 | 0.93 (0.78-1.12) | 0.473 |
| 3 to 4 | 0.58 (0.28-1.20) | 0.143 | 0.84 (0.62-1.14) | 0.270 |
| Living with elderly (Ref = 0) |  |  |  |  |
| 1 to 2 | 1.46 (0.79-2.69) | 0.227 | 1.04 (0.79-1.37) | 0.770 |
| 3+ | - | - | 0.90 (0.36-2.22) | 0.815 |
| Changed household incomes (Ref = Increased) |  |  |  |  |
| Decreased | 0.57 (0.31-1.06) | 0.078 | 0.82 (0.64-1.05) | 0.120 |
| No change | 0.78 (0.48-1.29) | 0.335 | 0.79 (0.63-0.99) | 0.037 |
| Concerns regarding household income (Ref = No) | 1.68 (1.11-2.53) | 0.014 | 1.28 (1.08-1.52) | 0.004 |
| Close friends or family infected with Covid (Ref = No) | 1.40 (0.94-2.08) | 0.099 | 1.25 (1.09-1.44) | 0.002 |
| Prior mental health condition (Ref = No/NA) | 1.53 (1.08-2.16) | 0.016 | 1.90 (1.64-2.20) | <0.001 |
| Resilience | 0.77 (0.60-0.99) | 0.043 | 0.80 (0.72-0.88) | <0.001 |
| No change to relationships with family or friends or colleagues (ref = No) | 0.44 (0.27-0.72) | 0.001 | 0.61 (0.50-0.75) | <0.001 |
| Closer relationship with parent/family (Ref = No) | 0.73 (0.47-1.15) | 0.181) | 0.99 (0.83-1.18) | 0.939 |
| Closer relationship with friends (Ref = No) | 1.44 (0.81-2.55) | 0.217 | 1.13 (0.92-1.39) | 0.251 |
| Worse relationship with partner (Ref = No) | 1.73 (1.06-2.84) | 0.029 | 1.48 (1.21-1.81) | <0.001 |
| Worse relationship with parent/family (Ref = No) | 1.17 (0.73-1.88) | 0.512 | 1.39 (1.15-1.68) | 0.001 |
| Worse relationship with friends (Ref = No) | 0.91 (0.59-1.38) | 0.644 | 1.60 (1.35-1.89) | <0.001 |
| Worse relationship with colleagues (Ref = No) | 1.15 (0.72-1.85) | 0.551 | 1.49 (1.22-1.83) | <0.001 |
| **WORKPLACE FACTORS** |  |  |  |  |
| Frontline category (Ref = ED) |  |  |  |  |
| ICU | 0.68 (0.31-1.50) | 0.340 | 0.93 (0.73-1.17) | 0.528 |
| Anaesthetics/Periop/Surgical | 0.93 (0.44-2.00) | 0.861 | 1.25 (0.98-1.61) | 0.072 |
| Medical specialty areas with general medicine, Hospital aged care | 1.13 (-0.691.85) | 0.615 | 1.10 (0.91-1.34) | 0.329 |
| Other (Paramedicine, radiology, pathology, maintenance, clerical, admin) | 0.65 (0.30-1.41) | 0.278 | 1.02 (0.73-1.44) | 0.903 |
| Primary care/Community, Residential or non-hospital aged care | 1.23 (0.65-2.30) | 0.526 | 1.03 (0.76-)1.40 | 0.846 |
| Working with COVID patients (Ref = No) | 1.16 (0.77-1.75) | 0.473 | 1.20 (1.02-1.40) | 0.025 |
| Received COVID Care training (Ref = No) | 1.09 (0.77-1.56) | 0.616 | 0.92 (0.80-1.05) | 0.220 |
| Received COVID PPE training (Ref = No) | 0.84 (0.51-1.37) | 0.474 | 0.93 (0.74-1.17) | 0.547 |
| Worried re transmitting Covid to family (ref = Not worried) |  |  |  |  |
| Neutral | 1.37 (0.64-2.93) | 0.412 | 1.01 (0.75-1.36) | 0.932 |
| Very worried | 3.15 (1.79-5.54) | <0.001 | 1.95 (1.55-2.45) | <0.001 |
| Worried about being blamed by colleagues (Ref = Neutral) |  |  |  |  |
| Strongly/somewhat disagree | 2.64 (1.40-4.99) | 0.003 | 0.96 (0.76-1.22) | 0.738 |
| Strongly/somewhat agree | 3.69 (2.20-6.22) | <0.001 | 1.78 (1.46-2.17) | <0.001 |

### Supplementary Table 4: Multivariate logistic regression of burnout - depersonalisation factors by rural and metropolitan locations

| **Burnout - Depersonalisation** | **Rural** |  | **Metropolitan** |  |
| --- | --- | --- | --- | --- |
|  | **OR (95%CI)** | **p** | **OR (95%CI)** | **p** |
| **PERSONAL FACTORS** |  |  |  |  |
| Female | 0.69 (0.45-1.05) | 0.086 | 0.62 (0.53-0.74) | <0.001 |
| Age group (Ref 50+) |  |  |  |  |
| 20-30 | 2.28 (1.13-4.63) | 0.022 | 2.81 (2.04-3.88) | <0.001 |
| 31-40 | 1.89 (1.06-3.38) | 0.031 | 1.84 (1.38-2.44) | <0.001 |
| 41-50 | 1.68 (1.03-2.75) | 0.039 | 1.60 (1.26-2.03) | <0.001 |
| State (Ref = Others) |  |  |  |  |
| Victoria | 0.68 (0.44-1.03) | 0.067 | 0.75 (0.60-0.93) | 0.009 |
| Profession (ref = Medical practitioner) |  |  |  |  |
| Pharmacist | 2.17 (0.91-5.17) | 0.079 | 1.07 (0.65-1.75) | 0.802 |
| Nursing | 0.88 (0.58-1.35) | 0.568 | 0.91 (0.77-1.07) | 0.255 |
| Allied Health/Paramedic/ Clinical Scientist | 0.80 (0.45-1.39) | 0.425 | 0.64 (0.51-0.80) | <0.001 |
| Other/Support/Leader/Clerical/Administrative | 0.81 (0.19-3.39) | 0.770 | 0.68 (0.30-1.53) | 0.351 |
| Years of experience since graduating (Ref = 0-5) |  |  |  |  |
| 6 to 10 | 1.09 (0.63-1.91) | 0.755 | 1.05 (0.86-1.29) | 0.609 |
| 11 to 15 | 0.97 (0.49-1.90) | 0.929 | 0.92 (0.71-1.19) | 0.535 |
| >15 | 0.79 (0.41-1.51) | 0.474 | 0.85 (0.64-1.13) | 0.263 |
| Living alone (Ref = No) | 1.00 (0.61-1.62) | 0.984 | 1.24 (1.02-1.52) | 0.035 |
| Living with children (ref = 0) |  |  |  |  |
| 1 to 2 | 0.86 (0.57-1.29) | 0.457 | 0.73 (0.61-0.88) | 0.001 |
| 3 to 4 | 0.61 (0.31-1.21) | 0.160 | 0.80 (0.60-1.08) | 0.147 |
| Living with elderly (Ref = 0) |  |  |  |  |
| 1 to 2 | 1.23 (0.66-2.27) |  | 0.67 (0.50-0.89) | 0.006 |
| 3+ | - | - | 0.91 (0.37-2.26) | 0.843 |
| Changed household incomes (Ref = Increased) |  |  |  |  |
| Decreased | 1.22 (0.66-2.25) | 0.534 | 0.80 (0.63-1.03) | 0.082 |
| No change | 1.10 (0.67-1.80) | 0.705 | 0.86 (0.70-1.07) | 0.176 |
| Concerns regarding household income (Ref = No) | 0.84 (0.56-1.28) | 0.419 | 1.04 (0.88-1.23) | 0.622 |
| Close friends or family infected with Covid (Ref = No) | 0.99 (0.67-1.46) | 0.951 | 1.08 (0.94-1.23) | 0.281 |
| Prior mental health condition (Ref = No/NA) | 0.87 (0.61-1.23) | 0.423 | 1.19 (1.03-1.37) | 0.022 |
| Resilience | 0.72 (0.56-0.92) | 0.009 | 0.78 (0.70-0.86) | <0.001 |
| No change to relationships with family or friends or colleagues (ref = No) | 0.80 (0.50-1.27) | 0.345 | 0.71 (0.58-0.86) | 0.001 |
| Closer relationship with parent/family (Ref = No) | 0.78 (0.50-1.23) | 0.286 | 0.93 (0.78-1.11) | 0.417 |
| Closer relationship with friends (Ref = No) | 1.02 (0.57-1.82) | 0.943 | 0.90 (0.73-1.11) | 0.339 |
| Worse relationship with partner (Ref = No) | 1.72 (1.06-2.79) | 0.029 | 1.26 (1.03-1.53) | 0.023 |
| Worse relationship with parent/family (Ref = No) | 1.01 (0.63-1.62) | 0.960 | 1.17 (0.97-1.41) | 0.100 |
| Worse relationship with friends (Ref = No) | 1.16 (0.76-1.76) | 0.490 | 1.24 (1.05-1.46) | 0.010 |
| Worse relationship with colleagues (Ref = No) | 1.26 (0.80-2.00) | 0.322 | 1.31 (1.07-1.59) | 0.008 |
| **WORKPLACE FACTORS** |  |  |  |  |
| Frontline category (Ref = ED) |  |  |  |  |
| ICU | 0.66 (0.32-1.34) | 0.246 | 0.68 (0.54-0.86) | 0.001 |
| Anaesthetics/Periop/Surgical | 1.04 (0.50-2.14) | 0.918 | 0.72 (0.57-0.91) | 0.007 |
| Medical specialty areas with general medicine, Hospital aged care | 0.78 (0.49-1.24) | 0.288 | 0.67 (0.55-0.81) | <0.001 |
| Other (Paramedicine, radiology, pathology, maintenance, clerical, admin,) | 0.47 (0.22-1.00) | 0.050 | 0.74 (0.53-1.03) | 0.078 |
| Primary care/Community, Residential or non-hospital aged care | 0.50 (0.27-0.93) | 0.028 | 0.55 (0.40-0.74) | <0.001 |
| Working with COVID patients (Ref = No) | 1.68 (1.14-2.47) | 0.009 | 1.21 (1.04-1.42) | 0.014 |
| Received COVID Care training (Ref = No) | 0.97 (0.68-1.36) | 0.846 | 0.88 (0.77-1.01) | 0.078 |
| Received COVID PPE training (Ref = No) | 0.64 (0.41-1.02) | 0.060 | 0.77 (0.62-0.96) | 0.018 |
| Worried re transmitting Covid to family (ref = Not worried) |  |  |  |  |
| Neutral | 0.90 (0.48-1.71) | 0.756 | 0.83 (0.63-1.09) | 0.174 |
| Very worried | 0.80 (0.48-1.31) | 0.375 | 1.10 (0.89-1.37) | 0.372 |
| Worried about being blamed by colleagues (Ref = Neutral) |  |  |  |  |
| Strongly/somewhat disagree | 0.87 (0.48-1.56) | 0.641 | 0.87 (0.70-1.09) | 0.224 |
| Strongly/somewhat agree | 1.84 (1.17-2.89) | 0.008 | 1.25 (1.03-1.51) | 0.021 |

### Supplementary Table 5: Multivariate logistic regression of burnout – emotional exhaustion factors by rural and metropolitan locations

| **Burnout – Emotional Exhaustion** | **Rural** |  | **Metropolitan** |  |
| --- | --- | --- | --- | --- |
|  | **OR (95%CI)** | **p** | **OR (95%CI)** | **p** |
| **PERSONAL FACTORS** |  |  |  |  |
| Female | 1.39 (0.89-2.19) | 0.152 | 1.08 (0.90-1.30) | 0.387 |
| Age group (Ref 50+) |  |  |  |  |
| 20-30 | 2.19 (1.00-4.83) | 0.051 | 2.21 (1.56-3.14) | <0.001 |
| 31-40 | 1.92 (1.03-3.55) | 0.039 | 1.44 (1.08-1.93) | 0.014 |
| 41-50 | 1.39 (0.84-2.29) | 0.199 | 1.49 (1.18-1.89) | <0.001 |
| State (Ref = Others) |  |  |  |  |
| Victoria | 0.65 (0.40-1.03) | 0.067 | 0.99 (0.78-1.25) | 0.904 |
| Profession (ref = Medical practitioner) |  |  |  |  |
| Pharmacist | 1.07 (0.40-2.89) | 0.892 | 1.65 (0.88-3.07) | 0.117 |
| Nursing | 1.04 (0.65-1.66) | 0.881 | 1.51 (1.26-1.80) | <0.001 |
| Allied Health/Paramedic/ Clinical Scientist | 1.07 (0.58-1.99) | 0.825 | 1.38 (1.08-1.78) | 0.011 |
| Other/Support/Leader/Clerical/Administrative | 3.82 (0.68-21.44) | 0.128 | 0.63 (0.29-1.37) | 0.241 |
| Years of experience since graduating (Ref = 0-5) |  |  |  |  |
| 6 to 10 | 1.18 (0.61-2.27) | 0.630 | 0.81 (0.63-1.04) | 0.093 |
| 11 to 15 | 1.09 (0.51-2.33) | 0.828 | 0.82 (0.61-1.11) | 0.205 |
| >15 | 1.47 (0.71-3.04) | 0.302 | 0.84 (0.61-1.14) | 0.261 |
| Living alone (Ref = No) | 1.32 (0.77-2.25) | 0.312 | 1.18 (0.94-1.49) | 0.151 |
| Living with children (ref = 0) |  |  |  |  |
| 1 to 2 | 1.00 (0.65-1.56) | 0.984 | 0.71 (0.59-0.87) | 0.001 |
| 3 to 4 | 1.05 (0.50-2.19) | 0.894 | 0.77 (0.56-1.06) | 0.109 |
| Living with elderly (Ref = 0) |  |  |  |  |
| 1 to 2 | 1.06 (0.58-1.95) | 0.846 | 0.84 (0.64-1.11) | 0.227 |
| 3+ | - | - | 1.05 (0.37-3.00) | 0.931 |
| Changed household incomes (Ref = Increased) |  |  |  |  |
| Decreased | 0.67 (0.34-1.31) | 0.237 | 0.94 (0.71-1.24) | 0.666 |
| No change | 0.71 (0.41-1.24) | 0.234 | 1.04 (0.82-1.32) | 0.769 |
| Concerns regarding household income (Ref = No) | 0.88 (0.56-1.37) | 0.565 | 1.17 (0.97-1.42) | 0.102 |
| Close friends or family infected with Covid (Ref = No) | 1.07 (0.70-1.63) | 0.770 | 0.94 (0.80-1.09) | 0.401 |
| Prior mental health condition (Ref = No/NA) | 1.74 (1.19-2.57) | 0.005 | 1.90 (1.59-2.28) | <0.001 |
| Resilience | 0.67 (0.50-0.89) | 0.006 | 0.65 (0.57-0.73) | <0.001 |
| No change to relationships with family or friends or colleagues (ref = No) | 0.74 (0.46-1.18) | 0.212 | 0.81 (0.66-1.00) | 0.048 |
| Closer relationship with parent/family (Ref = No) | 0.81 (0.50-1.31) | 0.387 | 0.94 (0.78-1.14) | 0.560 |
| Closer relationship with friends (Ref = No) | 0.80 (0.45-1.43) | 0.449 | 1.01 (0.80-1.26) | 0.962 |
| Worse relationship with partner (Ref = No) | 1.91 (1.00-3.67) | 0.051 | 1.59 (1.23-2.05) | <0.001 |
| Worse relationship with parent/family (Ref = No) | 1.45 (0.81-2.61) | 0.207 | 1.33 (1.04-1.69) | 0.021 |
| Worse relationship with friends (Ref = No) | 2.07 (1.25-3.46) | 0.005 | 1.48 (1.21-1.80) | <0.001 |
| Worse relationship with colleagues (Ref = No) | 1.33 (0.75-2.37) | 0.330 | 1.88 (1.45-2.45) | <0.001 |
| **WORKPLACE FACTORS** |  |  |  |  |
| Frontline category (Ref = ED) |  |  |  |  |
| ICU | 1.05 (0.48-2.30) | 0.898 | 0.92 (0.71-1.18) | 0.498 |
| Anaesthetics/Periop/Surgical | 1.43 (0.63-3.26) | 0.394 | 1.08 (0.83-1.41) | 0.568 |
| Medical specialty areas with general medicine, Hospital aged care | 1.29 (0.76-2.18) | 0.345 | 1.27 (1.02-1.57) | 0.030 |
| Other (Paramedicine, radiology, pathology, maintenance, clerical, admin,) | 1.11 (0.52-2.38) | 0.785 | 1.56 (1.06-2.30) | 0.025 |
| Primary care/Community, Residential or non-hospital aged care | 1.71 (0.87-3.33) | 0.119 | 1.28 (0.92-1.79) | 0.147 |
| Working with COVID patients (Ref = No) | 1.40 (0.90-2.16) | 0.134 | 1.08 (0.91-1.28) | 0.374 |
| Received COVID Care training (Ref = No) | 1.26 (0.87-1.82) | 0.225 | 0.95 (0.81-1.11) | 0.492 |
| Received COVID PPE training (Ref = No) | 0.75 (0.44-1.25) | 0.266 | 1.03 (0.81-1.31) | 0.810 |
| Worried re transmitting Covid to family (ref = Not worried) |  |  |  |  |
| Neutral | 0.53 (0.28-1.00) | 0.050 | 1.14 (0.87-1.50) | 0.342 |
| Very worried | 1.09 (0.66-1.81) | 0.731 | 1.85 (1.48-2.30) | <0.001 |
| Worried about being blamed by colleagues (Ref = Neutral) |  |  |  |  |
| Strongly/somewhat disagree | 1.37 (0.80-2.37) | 0.254 | 0.96 (0.77-1.21) | 0.761 |
| Strongly/somewhat agree | 1.98 (1.28-3.05) | 0.002 | 1.37 (1.12-1.67) | 0.002 |

### Supplementary Table 6: Multivariate logistic regression of burnout – personal accomplishment factors by rural and metropolitan locations

| **Burnout – Personal Accomplishment** | **Rural** |  | **Metropolitan** |  |
| --- | --- | --- | --- | --- |
|  | **OR (95%CI)** | **p** | **OR (95%CI)** | **p** |
| **PERSONAL FACTORS** |  |  |  |  |
| Female | 0.93 (0.59-1.49) | 0.772 | 1.08 (0.91-1.29) | 0.373 |
| Age group (Ref 50+) |  |  |  |  |
| 20-30 | 1.12 (0.53-2.35) | 0.765 | 0.93 (0.67-1.30) | 0.679 |
| 31-40 | 0.65 (0.36-1.19) | 0.161 | 0.97 (0.72-1.29) | 0.826 |
| 41-50 | 0.80 (0.48-1.34) | 0.394 | 0.89 (0.70-1.14) | 0.371 |
| State (Ref = Others) |  |  |  |  |
| Victoria | 0.98 (0.62-1.54) | 0.917 | 1.02 (0.81-1.29) | 0.870 |
| Profession (ref = Medical practitioner) |  |  |  |  |
| Pharmacist | 0.86 (0.33-2.27) | 0.763 | 0.50 (0.31-0.82) | 0.006 |
| Nursing | 0.69 (0.44-1.10) | 0.118 | 0.69 (0.58-0.82) | <0.001 |
| Allied Health/Paramedic/ Clinical Scientist | 1.24 (0.67-2.32) | 0.491 | 1.25 (0.98-1.61) | 0.074 |
| Other/Support/Leader/Clerical/Administrative | 0.58 (0.16-2.17) | 0.421 | 0.28 (0.13-0.60) | 0.001 |
| Years of experience since graduating (Ref = 0-5) |  |  |  |  |
| 6 to 10 | 1.40 (0.77-2.54) | 0.272 | 0.77 (0.62-0.95) | 0.015 |
| 11 to 15 | 1.20 (0.60-2.41) | 0.599 | 0.76 (0.58-1.00) | 0.049 |
| >15 | 1.57 (0.81-3.07) | 0.184 | 1.03 (0.77-1.39) | 0.835 |
| Living alone (Ref = No) | 1.23 (0.72-2.09) | 0.448 | 0.90 (0.74-1.11) | 0.342 |
| Living with children (ref = 0) |  |  |  |  |
| 1 to 2 | 0.97 (0.63-1.50) | 0.907 | 1.12 (0.93-1.35) | 0.234 |
| 3 to 4 | 1.39 (0.66-2.92) | 0.382 | 1.06 (0.77-1.44) | 0.733 |
| Living with elderly (Ref = 0) |  |  |  |  |
| 1 to 2 | 0.87 (0.47-1.62) | 0.670 | 1.06 (0.80-1.41) | 0.668 |
| 3+ | - | - | 0.65 (0.27-1.57) | 0.337 |
| Changed household incomes (Ref = Increased) |  |  |  |  |
| Decreased | 1.10 (0.58-2.08) | 0.770 | 0.78 (0.60-1.02) | 0.067 |
| No change | 1.34 (0.81-2.24) | 0.258 | 0.78 (0.62-0.99) | 0.042 |
| Concerns regarding household income (Ref = No) | 0.84 (0.55-1.29) | 0.431 | 0.88 (0.74-1.05) | 0.146 |
| Close friends or family infected with Covid (Ref = No) | 1.53 (0.99-2.36) | 0.056 | 1.11 (0.96-1.27) | 0.167 |
| Prior mental health condition (Ref = No/NA) | 1.37 (0.94-1.98) | 0.098 | 1.31 (1.12-1.53) | 0.001 |
| Resilience | 1.54 (1.19-2.00) | 0.001 | 1.83 (1.65-2.03) | <0.001 |
| No change to relationships with family or friends or colleagues (ref = No) | 0.98 (0.61-1.59) | 0.947 | 0.92 (0.75-1.12) | 0.404 |
| Closer relationship with parent/family (Ref = No) | 1.15 (0.71-1.86) | 0.572 | 1.29 (1.07-1.56) | 0.008 |
| Closer relationship with friends (Ref = No) | 0.78 (0.43-1.41) | 0.404 | 1.26 (1.00-1.59) | 0.047 |
| Worse relationship with partner (Ref = No) | 0.48 (0.29-0.78) | 0.003 | 0.88 (0.72-1.08) | 0.220 |
| Worse relationship with parent/family (Ref = No) | 1.13 (0.68-1.87) | 0.634 | 0.96 (0.79-1.17) | 0.696 |
| Worse relationship with friends (Ref = No) | 1.31 (0.84-2.06) | 0.235 | 0.96 (0.81-1.14) | 0.663 |
| Worse relationship with colleagues (Ref = No) | 1.02 (0.62-1.66) | 0.944 | 0.71 (0.58-0.87) | 0.001 |
| **WORKPLACE FACTORS** |  |  |  |  |
| Frontline category (Ref = ED) |  |  |  |  |
| ICU | 0.79 (0.36-1.74) | 0.561 | 1.09 (0.86-1.38) | 0.474 |
| Anaesthetics/Periop/Surgical | 0.70 (0.32-1.50) | 0.356 | 0.86 (0.67-1.10) | 0.220 |
| Medical specialty areas with general medicine, Hospital aged care | 0.98 (0.59-1.63) | 0.944 | 1.12 (0.92-1.37) | 0.259 |
| Other (Paramedicine, radiology, pathology, maintenance, clerical, admin,) | 0.64 (0.31-1.33) | 0.231 | 1.01 (0.72-1.42) | 0.955 |
| Primary care/Community, Residential or non-hospital aged care | 1.03 (0.54-1.97) | 0.930 | 1.21 (0.88-1.66) | 0.237 |
| Working with COVID patients (Ref = No) | 0.72 (0.47-1.10) | 0.128 | 0.79 (0.67-0.93) | 0.004 |
| Received COVID Care training (Ref = No) | 1.95 (1.35-2.83) | <0.001 | 1.18 (1.02-1.36) | 0.025 |
| Received COVID PPE training (Ref = No) | 1.03 (0.63-1.69) | 0.894 | 1.33 (1.07-1.66) | 0.010 |
| Worried re transmitting Covid to family (ref = Not worried) |  |  |  |  |
| Neutral | 1.03 (0.51-2.07) | 0.929 | 1.17 (0.89-1.56) | 0.266 |
| Very worried | 0.98 (0.57-1.67) | 0.930 | 1.07 (0.86-1.34) | 0.549 |
| Worried about being blamed by colleagues (Ref = Neutral) |  |  |  |  |
| Strongly/somewhat disagree | 1.84 (0.98-3.45) | 0.056 | 1.22 (0.97-1.53) | 0.094 |
| Strongly/somewhat agree | 0.93 (0.59-1.46) | 0.756 | 1.02 (0.84-1.24) | 0.818 |
